# *In silico* investigation of uncoupling protein function in avian genomes

**DOI:** 10.3389/fvets.2022.1085112

**Published:** 2023-01-19

**Authors:** Peymaneh Davoodi, Mostafa Ghaderi-Zefrehei, Mustafa Muhaghegh Dolatabady, Mohammad Razmkabir, Somayeh Kianpour, Effat Nasre Esfahani, Jacqueline Smith

**Affiliations:** ^1^Department of Animal Science, Faculty of Agriculture, Tarbiat Modares University, Tehran, Iran; ^2^Department of Animal Science, Faculty of Agriculture, Yasouj University, Yasouj, Iran; ^3^Department of Animal Science, Faculty of Agriculture, University of Kurdistan, Sanandaj, Iran; ^4^Department of Agriculture, Payam Noor University Tehran, Tehran, Iran; ^5^The Roslin Institute and Royal (Dick) School of Veterinary Studies R(D)SVS, University of Edinburgh, Edinburgh, United Kingdom

**Keywords:** avian uncoupling protein, nucleotide coding sequence, protein structure prediction, codon usage, pathway crosstalk

## Abstract

**Introduction:**

The uncoupling proteins (*UCPs*) are involved in lipid metabolism and belong to a family of mitochondrial anionic transporters. In poultry, only one *UCP* homologue has been identified and experimentally shown to be associated with growth, feed conversion ratio, and abdominal fat according to its predominant expression in bird muscles. In endotherm birds, cell metabolic efficiency can be tuned by the rate of mitochondrial coupling. Thus, *avUCP* may be a key contributor to controlling metabolic rate during particular environmental changes.

**Methods:**

This study aimed to perform a set of *in-silico* investigations primarily focused on the structural, biological, and biomimetic functions of *avUCP*. Thereby, using *in silico* genome analyses among 8 avian species (chicken, turkey, swallow, manakin, sparrow, wagtail, pigeon, and mallard) and a series of bioinformatic approaches, we provide phylogenetic inference and comparative genomics of *avUCP*s and investigate whether sequence variation can alter coding sequence characteristics, the protein structure, and its biological features. Complementarily, a combination of literature mining and prediction approaches was also applied to predict the gene networks of *avUCP* to identify genes, pathways, and biological crosstalk associated with *avUCP* function.

**Results:**

The results showed the evolutionary alteration of *UCP* proteins in different avian species. Uncoupling proteins in avian species are highly conserved trans membrane proteins as seen by sequence alignment, physio-chemical parameters, and predicted protein structures. Taken together, *avUCP* has the potential to be considered a functional marker for the identification of cell metabolic state, thermogenesis, and oxidative stress caused by cold, heat, fasting, transfer, and other chemical stimuli stresses in birds. It can also be deduced that *avUCP*, in migrant or domestic birds, may increase heat stress resistance by reducing fatty acid transport/b-oxidation and thermoregulation alongside antioxidant defense mechanisms. The predicted gene network for *avUCP* highlighted a cluster of 21 genes involved in response to stress and 28 genes related to lipid metabolism and the proton buffering system. Finally, among 11 enriched pathways, crosstalk of 5 signaling pathways including MAPK, adipocytokine, mTOR, insulin, ErbB, and GnRH was predicted, indicating a possible combination of positive or negative feedback among pathways to regulate *avUCP* functions.

**Discussion:**

Genetic selection for fast-growing commercial poultry has unintentionally increased susceptibility to many kinds of oxidative stress, and so *avUCP* could be considered as a potential candidate gene for balancing energy expenditure and reactive oxygen species production, especially in breeding programs. In conclusion, *avUCP* can be introduced as a pleiotropic gene that requires the contribution of regulatory genes, hormones, pathways, and genetic crosstalk to allow its finely-tuned function.

## 1. Introduction

Generalized homeostasis of energy expenditure and energy intake is essential for the best selection criteria in poultry, however, the regulatory mechanisms connecting feed intake, growth, and energy balance are still confusing. A growing body of literature implies avian Uncoupling Protein (*avUCP*) plays a key role in cell metabolism and adaptive thermogenesis. It thus may have the potential to be considered the missing link in the chain of whole-body energy homeostasis in chickens. The *avUCP* is a homolog with more than 70% protein sequence similarity with mammalian *UCP3* and *UCP2*, harboring two conserved regions of the mitochondrial carrier and ADP/ATP transporter translocase (1, 2). The *avUCP* gene was first identified in 2001 after screening a hummingbird skeletal muscle cDNA library (3). Subsequent studies in different avian species revealed a predominant expression of *avUCP* mRNA in skeletal muscle in chicken (*Gallus gallus*), king penguin (*Aptenodytes patagonicus*), and hummingbird (*Eupetomena macroura*) (1, 4–7). The interconnection of growth, oxidative stress, reproductive state, immunity, and feather coloration processes and their efficacy on thermo-regulation have been suggested by several studies (8–12).

For a long time, shivering thermogenesis has been known to be the main thermogenic mechanism in avian species (13, 14), although the evidence for the existence of adaptive mechanisms of heat production and non-shivering thermogenesis are currently growing (1). This thermogenesis mechanism can be boosted by increasing oxidative metabolic capacity along with the uncoupling of aerobic metabolism from ATP production. Also, previous studies support the involvement of *avUCP* in avian energy expenditure and adaptive thermogenesis (1, 15–18). Taouis et al. showed that early thermal conditioning in broiler chicks can instantly reduce body temperature and *avUCP* expression in the pectoral muscle, which may potentially improve the resistance to heat stress in broilers (15). In contrast, another study showed that diet-induced thermogenesis had no control over feed intake in layers and broilers and the expression of *avUCP* was not influenced by layer and broiler genotypes. Consequently, these findings led to the rejection of the hypothesis of the involvement of *avUCP* in diet-induced thermogenesis (19). A study in ducklings has verified that, in proportion to the degree of cold, the increase in metabolic heat production occurs in parallel with the upregulation of *avUCP* and higher mitochondrial oxidative phosphorylation, while no change in mitochondrial membrane conductance capacity occurred (20).

*avUCP* has also been suggested to be involved in cell metabolism. Several studies have identified polymorphism of the *avUCP* gene that is associated with fat metabolism, growth, feed intake, and exposure to abiotic stress conditions (2, 21, 22). Additionally, it was also revealed that the upregulation of *avUCP* can result in the down-regulation of reactive oxygen species (ROS) production in the skeletal muscle of fasted chickens (23). On the other hand, the *avUCP* expressed in glycolytic muscle fibers may be a passive transporter of pyruvate for ensuring a sustained balance between glycolysis and oxidative phosphorylation (24). Conversely, it was demonstrated that heat stress stimulates mitochondrial superoxide production in broiler skeletal muscle through the downregulation of uncoupling protein (25, 26). Another study showed that the expression of members of the beta-oxidation pathway and mitochondrial fatty acid transport were upregulated upon heat stress. However, the expression of *avUCP* did not control ROS production in heat-stressed chickens (18). Heat stress, by causing oxidative stress, impairs mitochondrial function by decreasing *avUCP* expression (27) which can further impair meat quality and increases glycolysis and intramuscular fat deposition (2, 28, 29). Additionally, the upregulation of *avUCP* and *avPGC-1*α together can help to reduce ROS accumulation and lipid oxidation in the skeletal muscles of birds (28, 30). Several distinct studies of *avUCP*, have demonstrated the regulation of *avUCP* expression and regulation of its putative function. Accordingly, thyroid hormones were reported to increase thermogenic capacity in the avian muscle and liver (31, 32). Moreover, uncoupling of sarco- endoplasmic reticulum calcium ATPase pump activity in muscle (33) and regulation of glycolysis are involved in controlling thermogenic processes in avian species. Some studies have implied that thermogenesis is controlled by thyroid hormone affecting *PPARGC1A* and *SLC25A4* gene expression in chickens (34, 35). However, triiodothyronine (T3) is reported to have a biphasic effect on *avUCP* expression (32). Another study investigating variations in *avUCP* expression, thyroid hormone metabolism, and heat production during cold exposure has reported a significant increase in body temperature, *avUCP* expression, *T3* level, renal outer-ring deiodination activity, and also increased thyroxine (*T4*) level, and hepatic inner-ring deiodination activity. Meanwhile, no significant differences in body weight and feed intake were reported in comparison with chickens reared in normal temperatures (36).

Moreover, it has been implied that *avUCP* gene expression is down-regulated by leptin hormone and up-regulated by pro-inflammatory cytokines *IL-6* and *TNF*α through modulation of *avUCP*-related transcription factors (*PPAR*s and *PGC-1*α) (32). Two-fold over-expression in gastrocnemius muscle, significant down-regulation, and no significant change were reported in *avUCP* mRNA expression through injection of thyroid hormone, methimazole, and insulin respectively (16). Additionally, selenium deficiency in broilers can cause a reduction in *avUCP* mRNA levels that results in oxidative stress, inflammation, and glyco-metabolism disorders (37).

Furthermore, in fat chickens with a higher fat diet, *avUCP* was significantly up-regulated, which could be correlated with the particular need for antioxidant pathways in muscle (38). Previous research has provided some evidence for the involvement of the beta-adrenergic system, *PPAR* transcription factors, and the AMP-activated protein kinase (*AMPK*) to control the expression of *avUCP* (39). Furthermore, oral use of D-aspartate resulted in a reduction in body temperature through the decline in *avUCP* mRNA expression in the breast muscle, which may be involved in reduced mitochondrial proton leaks and heat production (40).

There is also some evidence Oleuropein can also affect *avUCP* expression as well as genes related to mitochondrial oxidative phosphorylation and induce mitochondrial biogenesis in avian muscle cells. Oleuropeins can suppress mitochondrial superoxide production, through up-regulation of *avUCP* and manganese superoxide dismutase (41, 42). Therefore, the orexin system in avian muscle cells can regulate mitochondrial dynamics without affecting ATP synthesis (43). Evidence has also been presented that retinoic acid can activate the thermogenic function of *avUCP* in birds (44). Interestingly, “avian” is reported to be the only vertebrate lineage having just one *UCP* gene (45). Thus, the avian uncoupling protein seems to provide a unique opportunity to explore the functional activity and regulation patterns of *UCP*. We aimed, therefore, to investigate *avUCP*s in eight different avian species (chicken, turkey, swallow, manakin, sparrow, wagtail, pigeon, and mallard) through a wide range of comparative bioinformatics analyses to better understand the details of *avUCP*s, from their coding sequences to their functional consequences.

## 2. Methods

### 2.1. Coding sequence analysis

The nucleotide coding sequences and amino acid sequences of avian uncoupling proteins were downloaded from a dataset contained at https://figshare.com/. Sequence alignment of coding sequences (CDS) for eight avian species including chicken, turkey, swallow, manakin, sparrow, wagtail, pigeon, and mallard was conducted for determining the number of conserved, variable, parsimonious, and singleton sites in *avUCPs*. Moreover, nucleotide composition, GC content, codon frequency, and relative synonymous codon usage were obtained using MEGA11 software (46). The Codon Adaptation Index (CAI) for each of the studied avian species was estimated using the Markov model with 500 replications over the *avUCP* DNA sequence. As the reference set to calculate the CAI is important for interpretation, the codon usage table for each species (if it exists), from the codon usage database on the *CAIcal* server (http://www.kazusa.or.jp/codon/) was therefore utilized (47). The Relative Synonymous Codon Usage (RSCU) was calculated as follows:


(1) $$\begin{array}{l} RSCU_{i,j}=\frac{n_{i}x_{i,j}}{\sum_{j=1}^{n_{i}}x_{i,j}} \end{array}$$

Where *x*_*i*_ is the number of times the *ith* codon has been favored to be used for an amino acid, and *n* represents the number of synonymous codons for that amino acid.

### 2.2. Protein sequence analysis

Amino acid (a.a) composition, physio-chemical parameters and phylogenetic analysis of eight avian protein sequences were performed in QIAGEN *CLC* Genomics Workbench (RRID: SCR_011853) (48). Physio-chemical parameters including molecular weight, isoelectric point, extinction coefficient, instability index, aliphatic index, and grand average of hydropathicity, were determined for each *avUCP* protein sequence. The phylogenetic analyses were performed using the Neighbor-Joining method, Jukes-Cantor protein distance measure, and bootstrapping over 307 a.a of protein sequences of eight avian species in *CLC* Genomics Workbench (48). Entropy analysis was then carried out using *BioEdit* (49) to further determine variable and conserved sites and finally, by using the *Skylign* online tool, a positional logo of amino acid variability was constructed (50).

### 2.3. Protein structure prediction

The secondary structures of *avUCP*s were predicted by the *SOPMA* predictor (51). All *avUCP* sequences were submitted to the *Phyre2* web portal (http://www.sbg.bio.ic.ac.uk/~phyre2/html/page.cgi?id=index) as a batch file for tertiary structure prediction. After that, the *avUCP*s were modeled through four stages including homology detection, fold library scanning to predict secondary structure, loop modeling, and sidechain fitting (52). Structural evaluation and qualification were then performed using the *Swiss-Model* online tool (53).

### 2.4. Sequence-based gene ontology prediction

*PredictProtein* was used to predict Gene Ontology (GO) terms of cellular components, molecular function, and biological process for *avUCP* protein sequences (54). In this process, the distance between the input protein sequence and the closest annotated protein represents the reliability of GO prediction (54).

### 2.5. Interactive network prediction and gene-based enrichment analysis

By identifying genes related to *avUCP* from the literature, a list of genes was extracted according to Davoodi and Ehsani (55) for protein-protein network prediction. The list of most related-genes was provided through the retrospective review of previous studies on *avUCP* (5, 42, 56–71). Biomolecular network prediction and gene set enrichment analysis of networked genes were performed in *Cytoscape* (72) using *STRING* v11.5 (73, 74).

### 2.6. Pathway crosstalk prediction

Crosstalk prediction was applied using *XtalkDB* by querying pathways enriched for *avUCP* to predict which pairs of signaling pathways may interact to reach a conclusive understanding of biological pathways involved in the regulation of *avUCP* functions from a global view (75).

### 2.7. Datasets

The nucleotide coding sequences (CDS) and protein sequences of avian uncoupling protein (*avUCP*) from eight different avian species (chicken, turkey, swallow, manakin, sparrow, wagtail, pigeon, and mallard) were retrieved in FASTA format from the NCBI database and used for *in silico* analyses (Table 1).

**Table 1:** General information for *avUCP* genes in eight avian species extracted from the NCBI database.

**Species**	**Gene ID**	**Chromosome**	**Exon number**	**Transcript ID**
*Gallus gallus* (chicken)	373896	1	6	NM_204107.2
*Anas platyrhynchos* (mallard)	101794508	1	6	XM_005025525.4
*Chiroxiphia lanceolata* (lance-tailed Manakin)	116781978	2	7	XM_032677900.1
*Columba livia* (rock pigeon)	102092157	Unknown	8	XM_021285112.1
*Passer montanus* (eurasian tree sparrow)	120496512	Unknown	7	XM_039697035.1
*Hirundo rustica* (barn swallow)	120765208	2	7	XM_040089804.1
*Meleagris gallopavo* (turkey)	100303663	1	6	NM_001303164.1
*Motacilla alba alba (w*hite wagtail*)*	119699879	1	7	XM_038133918.1

### 2.8. Model of analysis

This research had an integrative pipeline but no unique statistical model. All parts of the pipeline are explained previously in each section.

## 3. Results

### 3.1. Coding sequence analysis

#### 3.1.1. Nucleotide composition

The CDS sequences were analyzed for nucleotide composition, GC content, conserved, variable, parsimony informative, and singleton sites. The CDS length in all selected birds consisted of 924 nucleotides. The number of conserved, variable, parsimony informative, and singleton sites were revealed as 684, 240, 145, and 95, respectively. Divergence details of GC content among the eight avian species are shown in Table 2. The “C” content in the coding sequences of *avUCP* in wagtail (2.5), mallard (1.0), sparrow (0.8), and manakin (0.1) was higher than that of “G,” however, the “G” content in turkey (1.8), chicken (1.2), swallow (0.6), and pigeon (0.3) was higher than that of the “C” content.

**Table 2:** GC content (%) of *avUCP* in eight different avian species.

	**C**	**G**	**C-1**	**G-1**	**C-2**	**G-2**	**C-3**	**G-3**	**GC**	**GC-1**	**GC-2**	**GC-3**
Chicken	32.5	33.7	26.6	35.1	26.0	23.1	44.8	42.9	66.1	63.8	49.7	87.6
Mallard	35.0	34.0	26.6	37.7	26.9	23.7	51.3	40.6	68.9	66.4	51.0	91.9
Manakin	32.0	31.9	26.0	37.7	26.0	22.1	44.2	36.0	64.0	65.8	48.7	79.9
Pigeon	34.1	34.4	26.0	37.3	27.3	23.4	49.0	42.5	68.5	65.2	51.2	91.6
Sparrow	34.2	33.4	25.3	37.0	26.3	23.4	51.0	39.9	67.6	65.3	51.0	90.8
Swallow	33.8	34.4	26.0	37.0	26.6	24.4	48.7	41.9	68.2	65.8	52.2	90.5
Turkey	31.8	33.7	26.3	35.1	26.0	23.1	43.2	42.9	65.5	63.6	49.8	85.9
Wagtail	35.8	33.3	25.6	37.7	26.3	23.4	55.5	39.0	69.2	66.6	50.9	94.5

#### 3.1.2. Codon usage analysis

The codon usage (CU) and relative synonymous codon usage (RSCU) values of *avUCP* coding sequence were calculated, then CU and RSCU patterns were obtained from the eight avian *avUCPs*. Generally, 64 combinations of 3-letter codons encode 20 different amino acids, thus showing codon redundancy. After excluding the three stop codons, 25 codons in pigeon, sparrow, turkey, wagtail, 24 codons in chicken, mallard, and swallow, and 23 codons in manakin *avUCP* were observed with an RSCU value higher than 1. Moreover, the numbers of unused codons (RSCU = 0) were as follows: chicken−13, mallard−14, manakin−8, pigeon−16, swallow−13, sparrow−15, turkey−11, and wagtail−18. The RSCU value of the codon CUG, which encodes leucine, was the highest in all selected avian species. By looking at codons with an RSCU >1 and examining their final bases, it was found that they ended, on average, with C (15), G (9), and roughly one U and A in the selected species. The highly preferred codons within *avUCP*, with their corresponding CU and RSCU values are presented in Table 3. As can be seen, all highly preferred codons, except the CAU (only in manakin), which encodes for histidine, end with a “C” or a “G.” In addition, these 25 highly preferred codons are responsible for encoding around 76% (in manakin) to 89% (in wagtail) of the total protein sequences of *UCP*. Moreover, the expected codon adaptation index (CAI) for retrieved CDSs of chicken, mallard, manakin, pigeon, sparrow, swallow, turkey, and wagtail were 0.707, 0.691, 0.712, 0.697, 0.695, 0.692, 0.709, and 0.696, respectively.

**Table 3:** The codon usage and relative synonymous codon usage of highly preferred codons for *avUCP* in eight avian species.

**Codon**	**Chicken**		**Mallard**		**Manakin**		**Pigeon**		**Swallow**		**Sparrow**		**Turkey**		**Wagtail**	
	**CU**	**RSCU**	**CU**	**RSCU**	**CU**	**RSCU**	**CU**	**RSCU**	**CU**	**RSCU**	**CU**	**RSCU**	**CU**	**RSCU**	**CU**	**RSCU**
AGC(S)	11	3.5	11	3.7	7	2.8	9	3.4	11	3.3	9	3.2	10	3.2	10	3.5
CGG(R)	8	2.1	6	1.6	7	2.0	8	2.1	12	3.0	6	1.6	8	2.1	9	2.4
CGC(R)	7	1.8	9	2.5	9	2.6	11	2.9	8	2.0	12	3.1	4	1.0	10	2.6
CUG(L)	25	4.4	20	3.8	20	3.2	20	3.8	22	3.9	21	3.9	24	4.4	20	3.8
CUC(L)	8	1.4	9	1.7	12	2.0	11	2.1	11	1.9	10	1.9	8	1.5	11	2.1
GCC(A)	18	2.3	22	2.4	21	2.2	20	2.3	30	3.2	28	3.0	20	2.6	31	3.4
GGG(G)	14	2.2	16	2.3	13	1.9	14	1.9	12	1.7	11	1.6	13	2.0	7	1.0
GGC(G)	9	1.4	11	1.6	9	1.3	9	1.2	11	1.6	11	1.6	7	1.1	18	2.7
GUG(V)	27	3.5	21	2.6	20	2.7	21	2.7	22	3.1	23	3.1	27	3.5	21	2.6
GUC(V)	4	0.5	10	1.3	7	0.9	9	1.2	5	0.7	6	0.8	4	0.5	10	1.3
ACC(T)	10	1.7	10	2.0	10	2.0	11	1.6	10	1.9	10	1.9	11	1.9	9	1.7
ACG(T)	9	1.6	9	1.8	3	0.6	11	1.6	7	1.3	8	1.5	10	1.7	8	1.5
CCC(P)	13	2.9	13	2.6	9	2.4	12	3.2	10	2.7	10	2.5	13	2.9	11	2.8
AUC(I)	10	2.5	11	3.0	8	2.4	10	3.0	10	3.0	10	2.7	11	2.8	8	3.0
AAG(K)	7	1.6	7	1.6	10	1.8	8	1.8	8	1.8	8	1.8	7	1.6	9	2.0
AAC(N)	8	1.8	7	2.0	6	1.3	8	2.0	6	1.7	7	1.8	6	1.3	8	1.8
CAG(Q)	11	1.8	9	1.5	13	1.9	13	2.0	11	2.0	12	2.0	12	2.0	11	1.8
CAC(H)	1	2.0	2	2.0	1	2.0	1	2.0	1	2.0	0	0.0	1	2.0	1	2.0
CAU(H)	0	0.0	0	0.0	0	0.0	0	0.0	0	0.0	1	2.0	0	0.0	0	0.0
GAG(E)	10	2.0	8	1.8	8	1.8	8	1.8	9	2.0	9	2.0	10	2.0	9	2.0
GAC(D)	7	1.4	11	2.0	6	1.1	10	1.8	9	1.6	10	1.8	8	1.6	10	1.8
UAC(Y)	9	1.6	11	2.0	9	1.6	8	1.5	10	1.8	11	2.0	9	1.6	11	2.0
UGC(C)	7	1.8	7	1.8	7	1.8	7	1.8	7	1.8	8	1.8	7	1.8	7	1.8
UUC(F)	11	2.0	9	1.8	9	1.8	10	1.8	7	1.4	9	1.8	10	1.8	9	1.8
UGG(W)	2	1.0	3	1.0	2	1.0	2	1.0	2	1.0	2	1.0	2	1.0	3	1.0
AUG(M)	7	1.0	6	1.0	7	1.0	6	1.0	10	1.0	11	1.0	8	1.0	11	1.0

### 3.2. Protein sequence analysis

#### 3.2.1. Amino acid compositions

**Figure 1:**
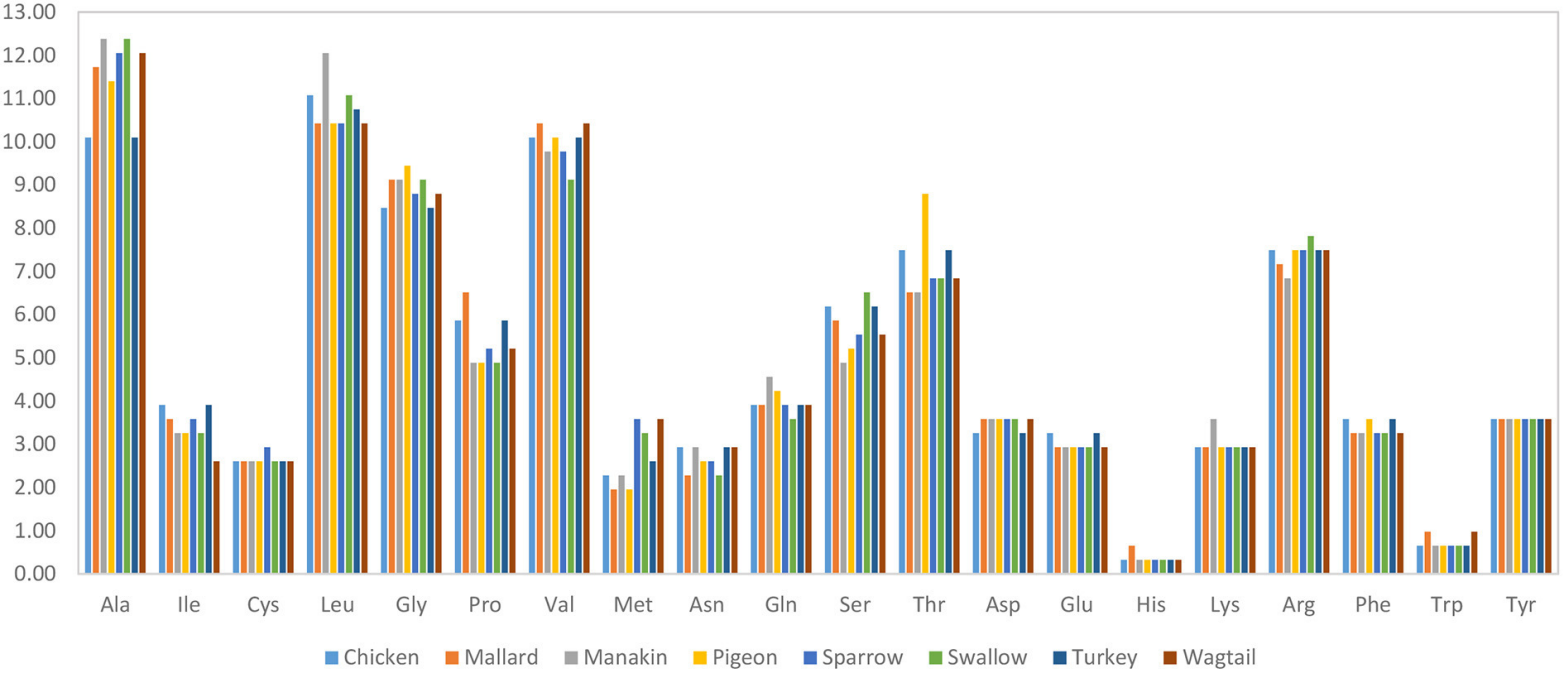
Amino acid composition of *avUCP* in different avian species.

The results of the protein sequence analysis of the eight avUCPs revealed a sequence length of 307 amino acids and the amino acid compositions in different birds are represented in Figure 1. Alanine, leucine, valine, and glycine have been observed at high frequency in all *avUCP*s, while histidine—a positively charged, and tryptophan—an aromatic amino acid, have been detected at the lowest frequency in all *avUCP*s. In contrast to the slight variation in amino acid usage among the eight studied *avUCP* sequences, tyrosine was the only completely constant amino acid among the *avUCP*s in all eight birds.

#### 3.2.2. Physio-chemical analysis

Physio-chemical parameters of uncoupling proteins including molecular weight, isoelectric point, aliphatic index, number of sulfur atoms, hydrophobicity, hydrophilicity, the percentage of negatively and positively charged amino acids, instability index, and grand average hydropathy of avian uncoupling protein in eight avian species are summarized in Table 4. The isoelectric point of *UCP*s ranged from 9.51 to 9.66. The lowest instability index (II) and the highest aliphatic index (AI) were observed in *UCP* of manakin. The molecular weight and the overall negatively/positively charged amino acids were nearly similar in all *avUCPs* in the current study.

**Table 4:** Physio-chemical parameters of uncoupling proteins from eight avian species.

**Species**	**MW (kDa)**	**IP**	**AI**	**S**	**H-phobic**	**H-philic**	**–R**	**+R**	**II**	**GRAVY**
Chicken	33.13	9.58	97.82	15	0.56	0.27	0.065	0.104	41.04	0.200
Mallard	32.81	9.51	96.58	14	0.58	0.25	0.065	0.101	39.74	0.209
Manakin	32.85	9.56	100.42	15	0.58	0.25	0.065	0.104	34.72	0.237
Pigeon	32.85	9.58	94.04	14	0.56	0.27	0.065	0.104	35.03	0.174
Sparrow	32.96	9.54	95.02	20	0.57	0.25	0.065	0.104	39.73	0.236
Swallow	32.84	9.66	94.72	18	0.57	0.25	0.065	0.107	38.59	0.215
Turkey	33.15	9.58	96.55	16	0.56	0.27	0.065	0.104	39.73	0.194
Wagtail	33.13	9.58	93.09	19	0.57	0.25	0.065	0.104	37.28	0.197

MW, Molecular weight; kDa, kilodalton; IP, Isoelectric point; AI, Aliphatic index; S, number of sulfur atom; H-phobic, Hydrophobic%; H-philic, Hydrophilic; –R, negatively charged; +R, positively charged; II, instability index; GRAVY, grand average hydropathy.

The atomic sulfur count varied from 14 (mallard, pigeon) to 20 (sparrow). Sulfur can be found in cysteine and methionine amino acids. Eight cysteine residues were observed in seven of the *avUCP*s but the protein sequence of sparrow contained nine cysteine residues. Furthermore, the hydrophobic methionine was variable among *avUCP*s, which could be a source of variability in atomic sulfur count among *avUCP* sequences in the studied species.

A protein with an II smaller than 40 is considered stable, and proteins with an II above 40 can be considered somehow unstable (76, 77) and in the current study, the highest II was observed in *UCP* of chicken. Also, protein sequences with a GRAVY index above 0 are more likely to be hydrophobic and all studied proteins were revealed to have more hydrophobic regions.

#### 3.2.3. Entropy analysis

**Figure 2:**
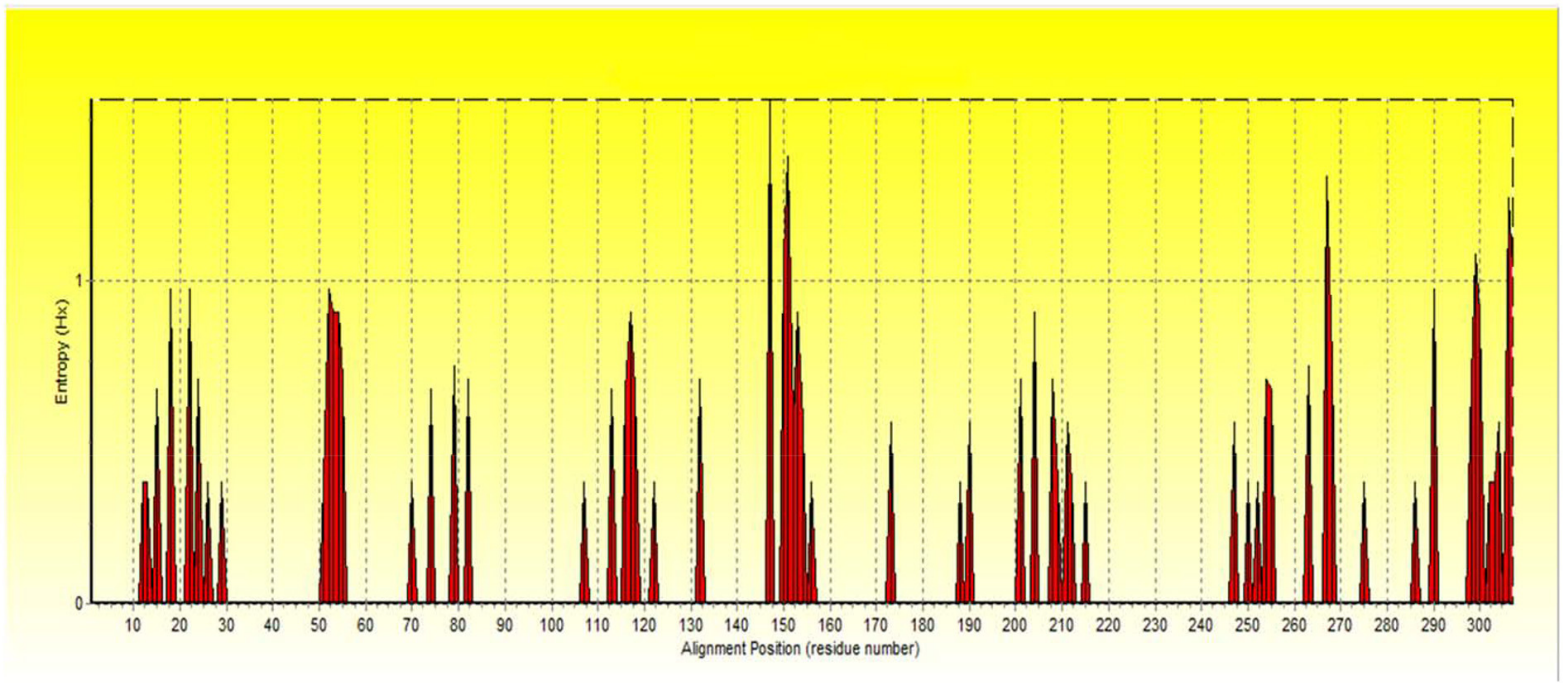
The Shannon entropy plot for alignment of proteins.

**Figure 3:**
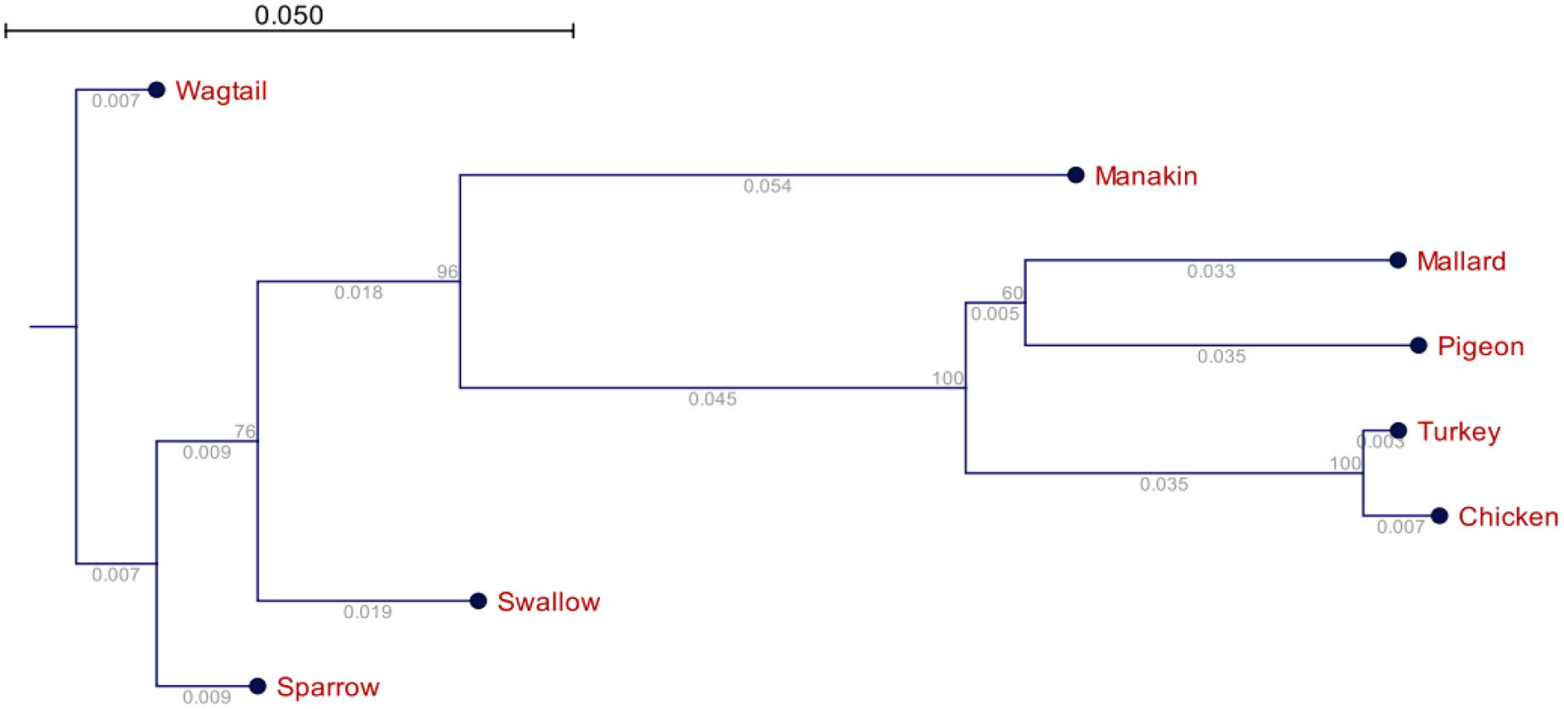
Phylogenetic relationships of the *avUCP*s in eight avian species (the numbers below the lines represent the branch size, and the numbers above the lines represent the bootstrap value).

For more evaluation of the status of amino acids in the *avUCP* protein sequences, entropy measures for each position were estimated using *BioEdit* (49) and visualized by the Shannon entropy plot, as shown in Figure 2. The estimated entropies of aligned sequences ranged from 0 to 1.56, the average entropy was estimated as 0.133 and a total of 274 positions displayed an entropy of 0. Seven positions revealed entropy values higher than 1 (147, 151, 267, 306, 299, 150, and 307). Among these, 147th and 151st positions showed the highest entropy of 1.56 and 1.39, respectively. Also, eight regions with a length of more than 10 amino acids (1 → 11, 30 → 50, 56 → 70, 83 → 107, 133 → 146, 157 → 172, 174 → 187, 216 → 246) have represented entropy of zero, indicating totally conserved regions in the examined *avUCP* protein sequences. Moreover, any region with more than 10 consecutive amino acids with an average entropy of >1 was not observed. To provide a compact representation of the most variable sites with the highest entropy in *avUCP* sequences among these eight species, the *Skylign* online tool (50) was used to make a positional logo showing amino acid variability (Figure 3).

##### 3.2.3.1. Phylogeny analysis

**Figure 4:**
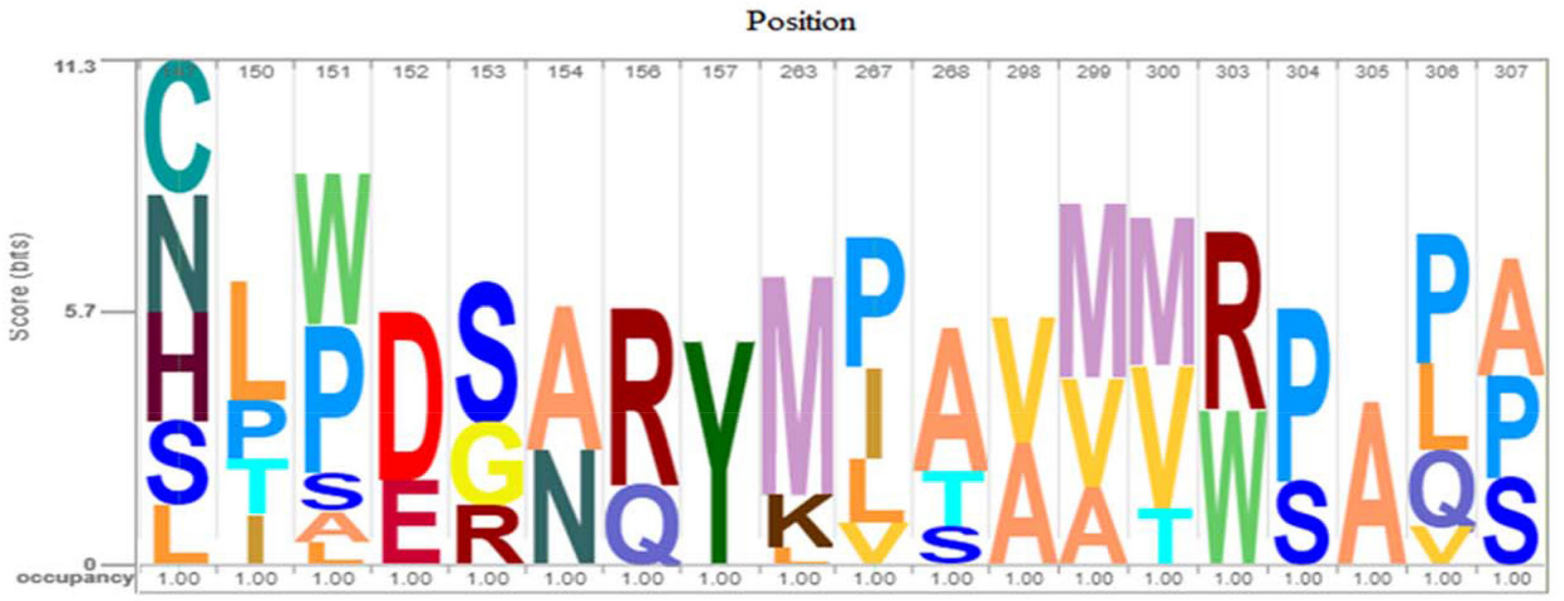
Logo of *avUCP* protein alignments (most variable sites with high entropy) among eight avian species. The height of the bar of letters (amino acids) displays the conservation at that position and the height of each letter within a bar is determined based on the frequency of that letter in that position.

The phylogenetic relationship of the *avUCP*s in the eight examined avian species is depicted in Figure 4. A neighbor-joining tree was derived from the multiple protein sequence alignment. Phylogenetically, the longest distance was detected between chicken and wagtail, in contrast chicken and turkey showed the closest phylogenetic relationship which is in agreement with comparative results of amino acid component, codon usage pattern and physio-chemical parameters among *avUCP*s of mentioned birds.

### 3.3. Protein structure prediction

**Figure 5:**
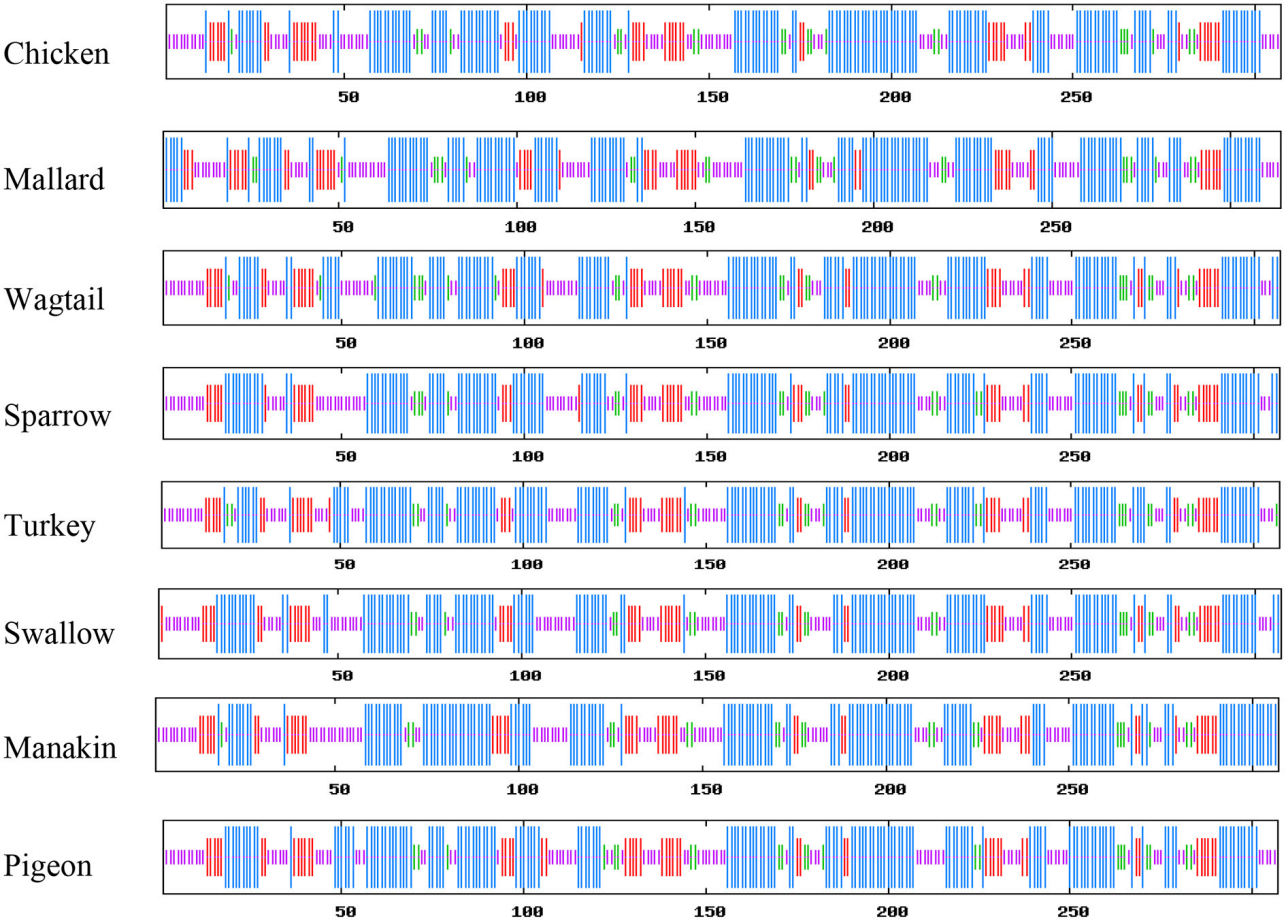
Comparative view of the secondary structure of *avUCPs* in eight avian species (Blue, Alpha helix; Purple, Random coil; Red, Extended-strand; Green, Beta turn).

**Figure 6:**
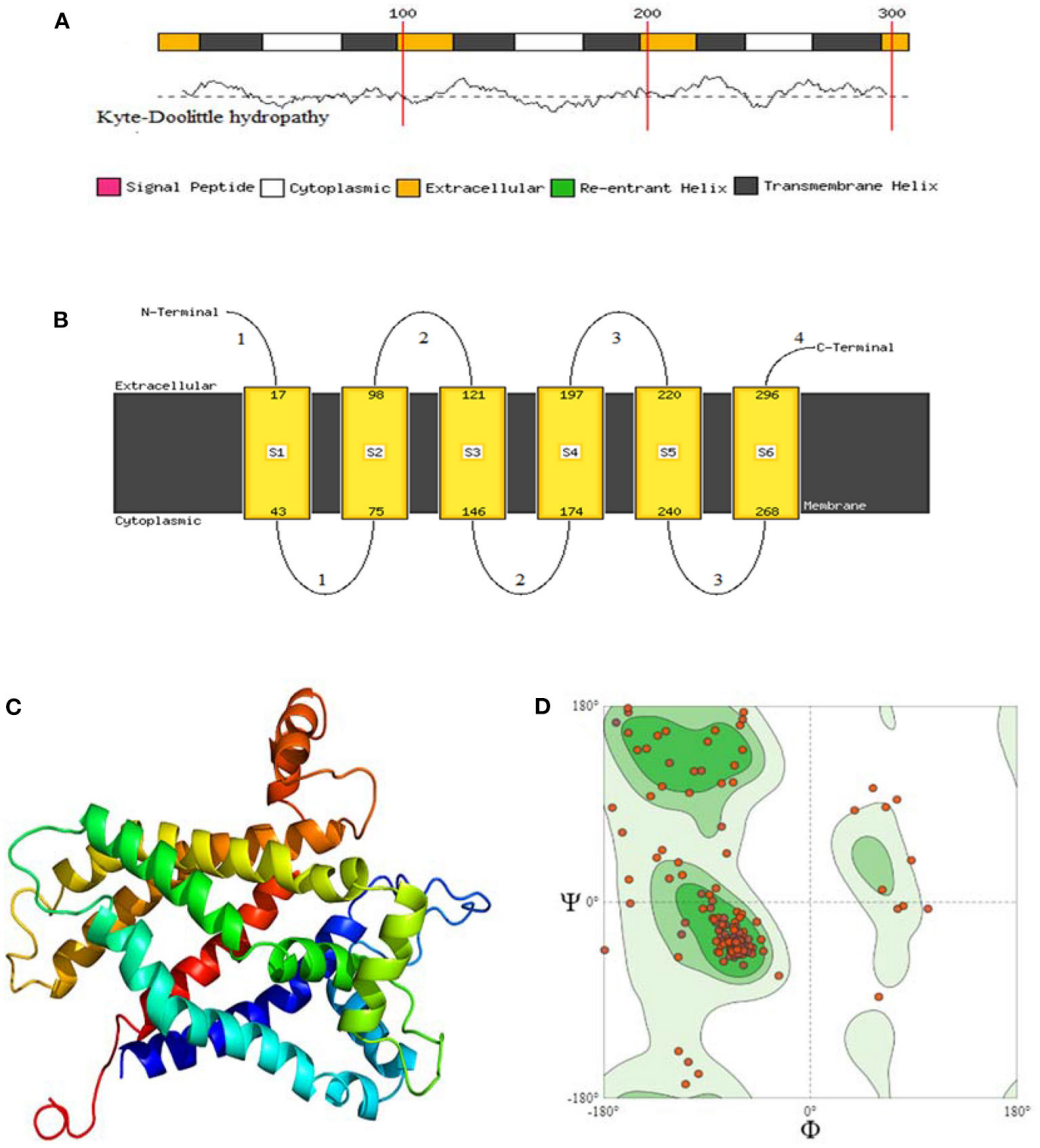
**(A)** Membrane helix prediction with support vector machines with Kyte-Doolittle hydropathy plot. **(B)** Schematic figure of predicted extracellular, cytoplasmic, and transmembrane helices. **(C)** Top 3D model of avian *UCP* [Model dimensions (Å): X: 53.347, Y: 74.255, Z: 50.565, Image colored by the rainbow N → C terminus]. **(D)** Ramachandran plot with 88.46% favored. A dihedral angle of a protein is the internal angle of polypeptide backbone at which two adjacent planes meet. The conformation of the backbone can be described by two dihedral angles per residue, because the backbone residing between two juxtaposing Cα atoms are all in a single plane. These angles are called ϕ (phi) which involves the backbone atoms C-N-Cα-C, and ψ (psi) which involves the backbone atoms N-Cα-C-N.

SOPMA was used to determine the percentage of α-helix, β-sheets, turns, and random coils to predict the secondary structure of the selected *avUCP* sequences through a neural network approach (51). The schematic predicted secondary structures of *avUCP*s in the eight avian species are shown in Figure 5. Because the secondary and tertiary structures of the protein are completely influenced by its primary structure, any differences in amino acid sequences can potentially modify the secondary and tertiary structures. Within the eight *avUCP* proteins, slightly different percentages of α-helical and β-turn formations were derived. The average contribution of alpha-helices, extended-strands, beta-turns, and random coils were calculated as 46.60 ± 1.04, 15.87 ± 0.58, 7.42 ± 0.65, and 30.08 ± 0.95% among the eight *avUCP* proteins, respectively. The level of alpha-helical structure was determined higher than other secondary structures.

he protein structure has been predicted by three phyre2, PredictProtein, and SOPMA software, and the final structure evaluation was performed by SWISS_MODEL for checking the clashing score, Ramachandran residues favored, and rotamer outliers. By using the *Phyre2* web portal (52), through the homology detection method, three-dimensional structures of the eight *avUCP*s alongside potential extracellular, cytoplasmic, and transmembrane helixes were predicted. In the protein structure, 6 transmembrane, 4 extracellular, and 3 cytosolic regions were predicted for the *avUCP*s. The two C-terminal and N-terminal segments were considered extracellular regions. Moreover, both hydrophobic and hydrophilic regions can be seen throughout the *avUCP* sequence. Accordingly, six areas with positive hydropathy are likely to represent transmembrane helixes, which indicates *avUCP* can function as a transmembrane protein (Figure 6A). Meanwhile, areas with negative hydropathy show that these regions can form the extracellular part of *avUCP* (Figure 6A).

Ultimately, among the eight *avUCP*s, the predicted structure with the highest sequence identity (71%), alignment coverage (94%), interface similarity (51%), and confidence (100%) is illustrated in Figures 6A–C. The nuclear magnetic resonance molecular fragment replacement approach was applied to re-specify protein structure using the *Swiss-Model* web tool. The local and global model quality was then specified from Ramachandran analysis for the predicted tertiary structure of *avUCP* (Figure 6D). The final structure evaluation resulted in a clash score (the number of serious clashes per 1,000 atoms) of 90%, Ramachandran residues favored−88.46%, Ramachandran outliers−3.50%, rotamer outliers−8.09%, 0.01 bad angles, and two C-beta deviations.

### 3.4. Sequence-based gene ontology prediction

**Figure 7:**
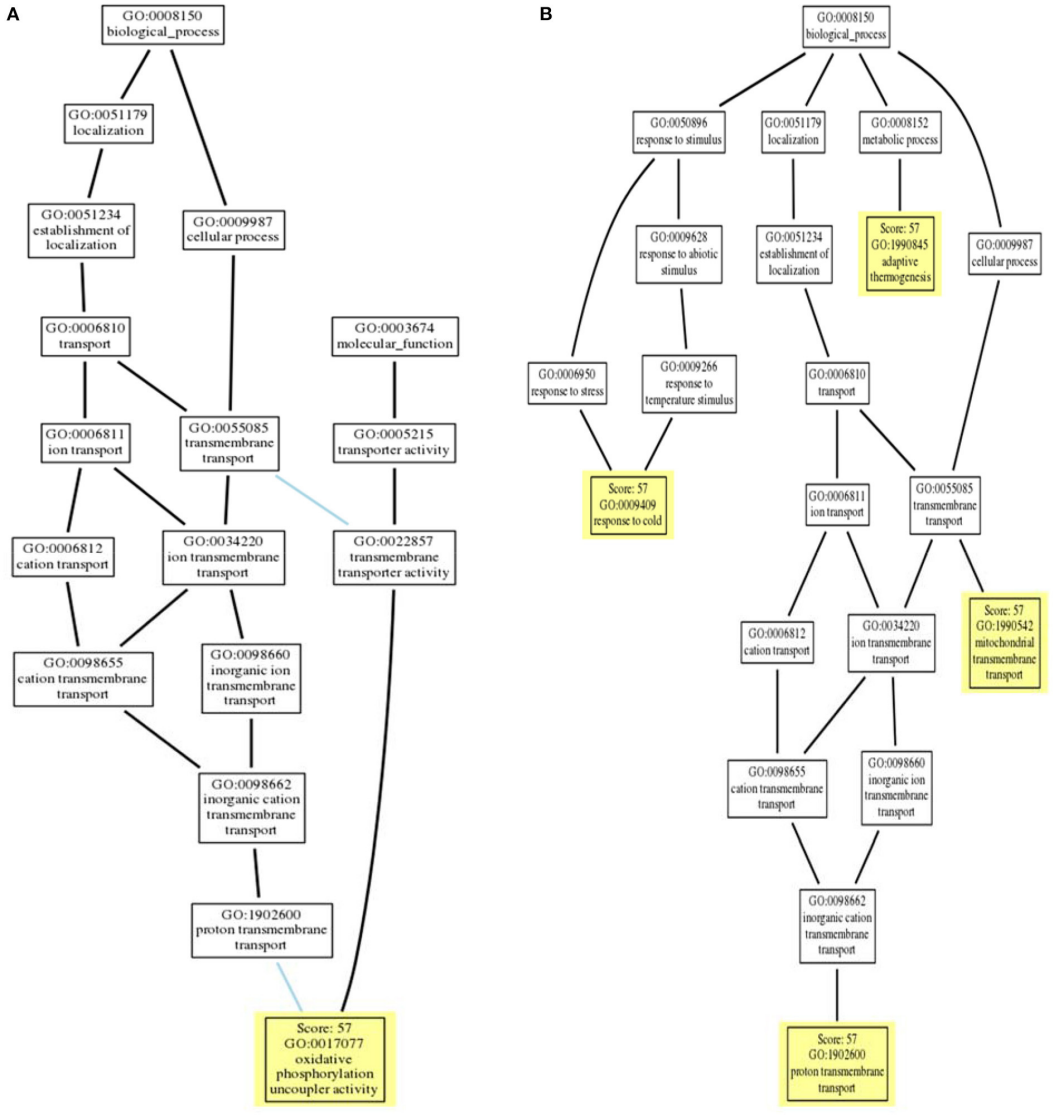
**(A)** Molecular function ontology tree and **(B)** biological process ontology tree (locations of PREDICTED terms are highlighted in yellow with respect to inferred terms).

Gene Ontology (GO) terms associated with *avUCP* were predicted using deep learning embedding through the *PredictProtein* online tool (54). For this reason, the distance between the input *avUCP* protein and the closest annotated protein was represented as the reliability of GO prediction. The GO trees of *avUCP* are depicted in Figure 7. Consequently, four biological processes including “mitochondrial transmembrane transport,” “proton transmembrane transport,” “adaptive thermogenesis,” and “response to cold” were predicted with 57% reliability. Also, three cellular components including “mitochondrion,” “mitochondrial inner membrane,” and “integral component of the membrane,” along with one molecular function of “oxidative phosphorylation uncoupler activity” were also anticipated with 57% confidence.

### 3.5. Interactive network prediction and gene set enrichment analysis

**Figure 8:**
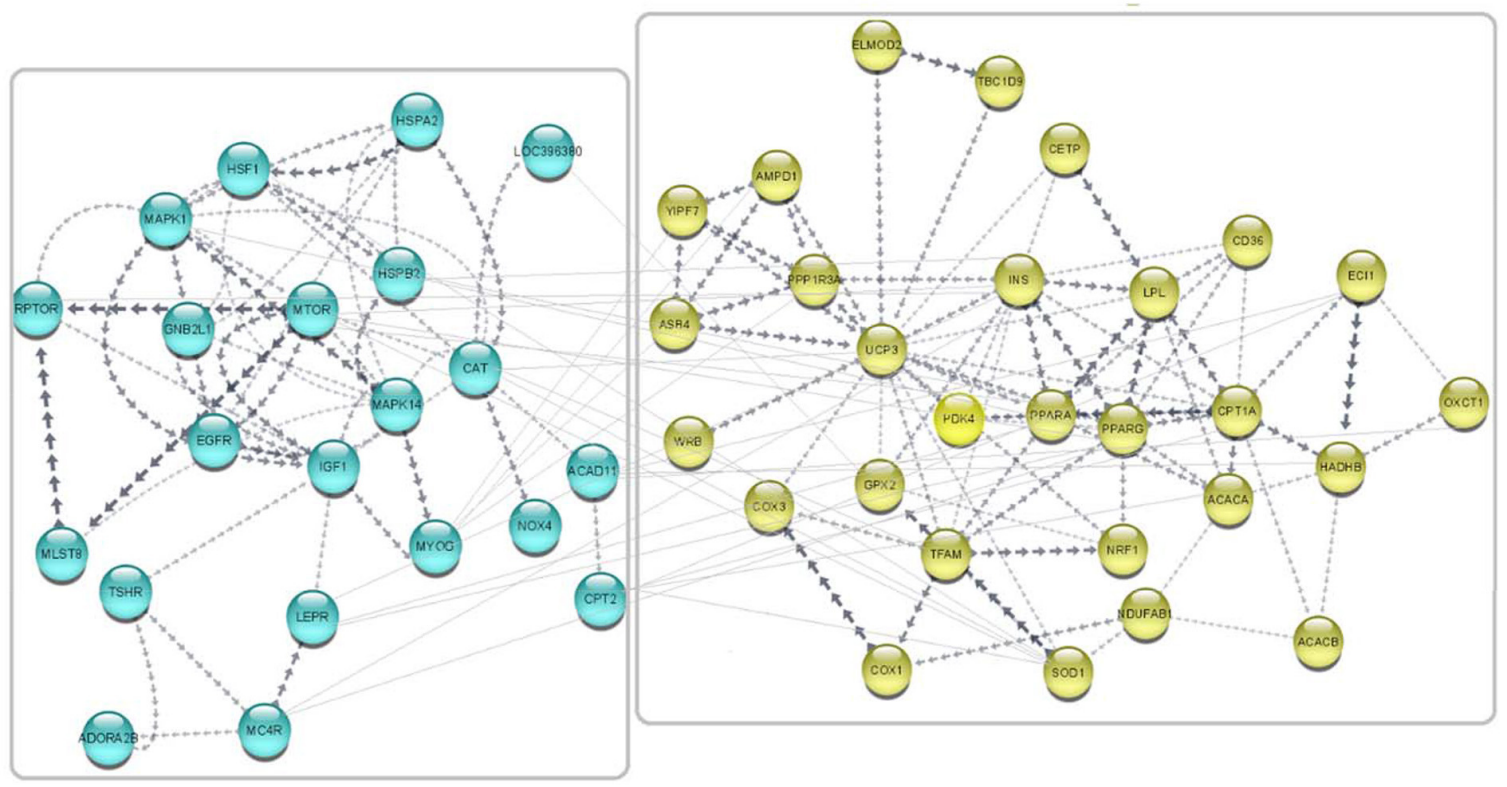
Predicted network for the regulatory and collaborative genes with *avUCP*. Separate boxes show clusters. Blue nodes represent genes involved in response to stress (cold and free radicals) (cluster 1). Yellow nodes represent genes involved in lipid metabolism and proton buffering system (cluster 2).

Gene network was predicted in *Cytoscape* software (3.9.1) using the embedded *STRING* app, and clustering was performed by the K-means method (72). A total of 49 published *avUCP* related-genes were used as input for network prediction and resulted in highly orchestrated interactions among genes. This network is divided into two clusters with the highest confidence: cluster one containing 21 genes involved in response to stress, and cluster two containing 28 genes involved in lipid metabolism and proton buffering system. The predicted network is shown in Figure 8. GO analysis on these networked genes highlighted several biological processes including the fatty acid metabolic process (GO:0006631), response to chemical (GO:0042221), cellular response to chemical stimulus (GO:0070887), oxidation-reduction process (GO: 0055114), fatty acid beta-oxidation (GO: 0006635), and regulation of fatty acid metabolic process (GO:0019217) as being enriched (FDR < 0.01). Furthermore, three cellular components including “mitochondrion,” “TOR complex1,” and “mitochondrial membrane,” were enriched (FDR < 0.01). The PPAR signaling pathway, adipocytokine signaling, FoXO signaling, mTOR signaling pathway, insulin signaling pathway, MAPK signaling pathway, fatty acid degradation, fatty acid metabolism, GnRH signaling pathway, ErbB signaling pathway, and oxidative phosphorylation were also indicated as enriched KEGG pathways for the *avUCP* gene network (FDR < 0.05).

### 3.6. Pathway crosstalk

**Figure 9:**
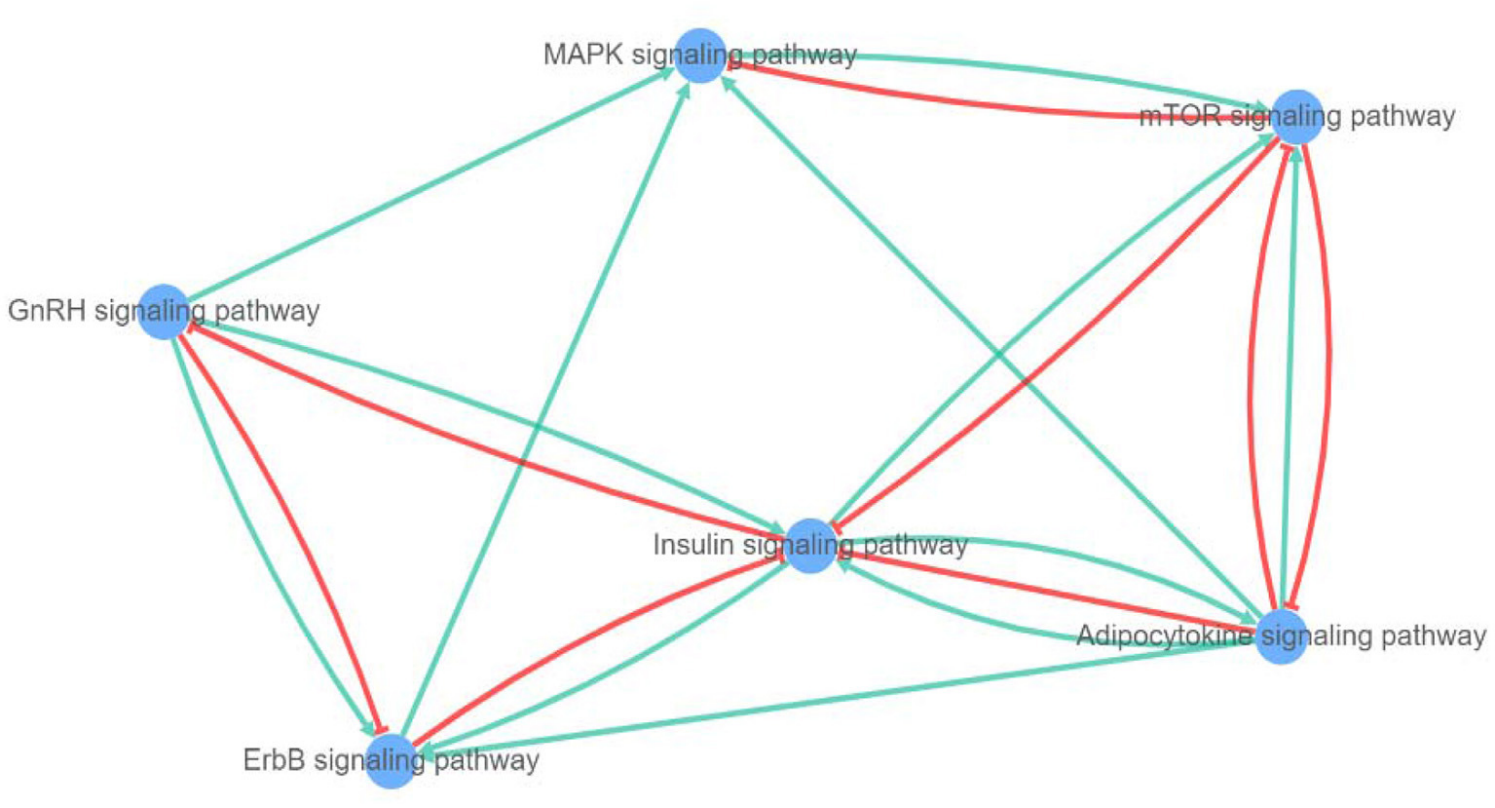
Network of crosstalk among enriched pathways (blue lines show activation and red lines represent inhibition effect).

After a query of enriched pathways, we used XtalkDB (75) to predict which pairs of signaling pathways may crosstalk with each other. The six enriched pathways were predicted to be involved in a network of crosstalk which is depicted in Figure 9. In detail, adipocytokine signaling was revealed to have an activation effect on MAPK, insulin, and ErbB signaling pathways, and both activation and inhibition effects on the mTOR signaling pathway.

The mTOR signaling pathway is predicted to act as an inhibitor for both MAPK and insulin signaling pathways. Among these, the MAPK showed only an activation effect on the mTOR pathway. Moreover, the insulin signaling pathway is anticipated to induce adipocytokine, mTOR, and ErbB signaling pathways, alongside inhibited GnRH signaling pathway. The GnRH signaling pathway is shown to be involved in activation of adipocytokine and MAPK signaling pathways and both negative and positive crosstalk with ErbB signaling pathway. In addition, the ErbB signaling pathway can activate and silence MAPK and insulin signaling pathways, respectively.

## 4. Discussion

### 4.1. Coding sequence analysis

#### 4.1.1. Codon usage analysis

In the investigation of codon usage, the RSCU value is a ratio between the occurrence frequency of a certain codon and the expected usage frequency for codons (78). Codons encoding amino acids of *avUCP* with RSCU values higher than 1.0 represent positive codon usage bias and codons with RSCU values lower than 1 display negative codon usage bias. Moreover, codons with RSCU = 0 display unfavorable codons. According to the results, the *avUCP* gene obviously prefers codons with “C” and “G” in the third position over the other bases. It can be concluded that almost all highly preferred codons, except the CAU codon (only in manakin), which encodes for histidine, end with a “C” or a “G.”

However, the mechanisms of inducing codon biases remained an open question. It can be attributed to the expression level of genes, selective pressure, evolutionary trend, phylogenetic relations of organisms, and genetic drift (79–81).

Moreover, the CAI values of 1 and 0 refer to the species in which only the most frequent codons are used, and species using the least frequent codons. Although manakin had the lowest GC ratio among the studied birds, it revealed the highest CAI, indicating that it uses 71.2% of the frequently used codons. Manakin uses most of the synonymous codons (53/61 codons) to encode *avUCP* so that it has the lowest number of unused codons (8). Interestingly, manakin is the only species among the selected birds which breeds in tropical forests. Therefore, the observed differences in manakin may be a result of adaptation to tropical conditions. Furthermore, the use of a comparatively wide range of synonymous codons alongside the use of frequently-used codons can be regarded as an evolutionary variation to achieve efficient translation in relatively important functional genes.

### 4.2. Protein sequence analysis

In protein sequence and physio-chemical analysis, a slight variation in amino acids and physio-chemical parameters among the eight studied *avUCP* proteins was detected. Tyrosine was the only amino acid showing a constant level in all avian species. The range of isoelectric points of *UCP*s implies that *UCP*s can be membrane proteins. As the pH of the intermembrane space and the mitochondrial matrix is about 7.0 and 8.0, respectively, thus *UCP*s carry an electrical charge in that region. Additionally, since aliphatic side chains like alanine, leucine, and valine determine the aliphatic index (AI) of a protein, *avUCP*, which shows a high content of these amino acids, could be considered thermostable. The highest AI of 100.42 was observed in manakin *avUCP* which may illustrate the importance of *avUCP* stability specifically in manakin.

The variation of atomic sulfur count from 14 to 20 among the eight *avUCP* proteins illustrates another significant difference among avian species. Sulfur can be found in cysteine and methionine amino acids. Nine cysteine residues were observed in *avUCP* in sparrow but the protein sequence of the others contained eight cysteine residues. Furthermore, the hydrophobic methionine was variable among *avUCP*s, which could be the second source of variability in atomic sulfur count among *avUCP* sequences in the studied species. Sulfur-containing amino acids are responsible for stronger connections than aliphatic and aromatic amino acids. Thus, they can provide a more sustainable 3D structure representing functional specificity in membrane proteins by creating a disulfide bond (77, 82). The percentage of hydrophobic and hydrophilic amino acids in the studied sequences were very close to each other indicating that these chemical characteristics play important roles in the encoded *avUCP* protein. The higher potential of hydrophobicity according to GRAVY indices above 0 is another appropriate state for transmembrane proteins. If a protein plays an important functional role, the state of its hydrophobicity and hydrophilicity will remain stable as much as possible, for the conservation of its function. Also, the stability of positively and negatively charged amino acids among all studied *avUCP*s may imply the effectiveness of charged regions of this protein which needs to be conserved among birds.

#### 4.2.1. Entropy analysis

The positions of aligned sequences in the entropy plot can correlate with the structural and chemical characteristics of certain amino acids and their influence on the function of *avUCP*s. Therefore, regions that contain residue positions with low entropy are more likely to be involved in the functional sites of *avUCP* (83, 84). Moreover, the absence of any region with more than 10 consecutive amino acids with an average entropy of >1 can be concluded as evidence of conservation in *avUCPs*.

### 4.3. Protein structure prediction

Because of the high frequency of alanine, leucine, valine, and glycine, and the low frequency of histidine and tryptophan in all *avUCP*s, the level of alpha-helical structure was determined higher than in other secondary structures. In agreement with our result, it is already known that transmembrane regions of proteins contain a high level of alpha-helices devoid of polar amino acids, while extracellular and cytoplasmic regions of the protein are usually enriched with polar amino acids like tyrosine and tryptophan (77).

The Ramachandran parameters of predicted protein can display the statistical distribution of the combinations of torsional *Phi* and *Psi* angles in the *avUCP* protein structure (85). Moreover, rotamers in protein structure imply conformational isomers of amino acid residues in the sidechain of *avUCP*, therefore, rotamer outliers display conformations that drop outside the reference (86). Also, C-beta deviation can reflect misfit conformation and inconsistency between sidechain and protein backbone that can be used for structure validation (87). In the current structural modeling for *avUCP*, the two C-beta deviations have resulted from valine and proline amino acids in positions 56 and 50, respectively, which were predicted to be in the cytoplasmic region of *avUCP*.

Hence, in addition to regulatory hormones and elements, different innate parameters can affect gene expression patterns of any pleiotropic genes like *avUCP*, with those parameters including, codon usage, GC content, CpG dinucleotide content, splicing sites, CpG islands, mRNA secondary structure, coding sequences (CDS), ribosomal binding sites, stimulators, the expression of other genes, along with environmental conditions (78, 88). For example, previous research has revealed cysteine residues of *UCP*s can be glutathionylated (89–92). They suggest that reactive oxygen species and glutathionylation can regulate non-phosphorylating respiration. Mailloux et al. (93) have identified Cys^25^ and Cys^259^ as the probable glutathionylation sites on *UCPs* (93). Interestingly, in the current study Cys^25^ and Cys^257^ were determined as conserved sites and predicted to be located in transmembrane and cytoplasmic regions of avUCP, respectively.

### 4.4. Sequence-based gene ontology prediction

The sequence-based predicted biological processes of “mitochondrial transmembrane transport,” “proton transmembrane transport,” “adaptive thermogenesis,” and “response to cold” for *avUCP* are congruent with results from previous studies. In this regard, some studies support the involvement of *avUCP* in avian energy expenditure and adaptive thermogenesis (1, 15–18). Additionally, it should be mentioned that cold acclimation can not only induce fatty acid-mediated uncoupling of oxidative phosphorylation processes but also increases the rate of ADP and Pi concentrations, along with ATP synthesis in the mitochondria of chicken skeletal muscle, which seems to be a counterproductive occurrence in response to cold stress condition in birds (34). Moreover, Ueda et al. reported a correlation between uncoupling and both exogenous and endogenous fatty acids in the mitochondria of chicken skeletal muscle during cold temperatures (94). Another study conducted on king penguins showed that superoxide activates the proton transport of mitochondria and GDP inhibits the transport of the superoxide-activated-proton, demonstrating that *avUCP* mediates mitochondrial proton transport but plays no role in the basal proton leak (6). Because thermogenic hormones have an induction effect on *avUCP* expression, the involvement of avUCP in avian thermogenesis can be concluded (16).

### 4.5. Interactive network prediction and gene-based enrichment analysis

Through the network of gene-gene interactions, centralized by *avUCP* and using previously recognized *avUCP-* related genes (5, 34, 42, 56–71, 94), we have found two major clusters of genes pointing to the overall functionality of response to stress and lipid metabolism/proton buffering. Our well-categorized findings are in agreement with the outputs of other studies. Previously conducted studies revealed the predominant presence of *avUCP* protein in skeletal muscles (pectoral, glycolytic fibers) (90) alongside recognizing the alteration in expression pattern during different physiological states [cold stress (10, 12, 34, 58, 90), heat stress (12, 29, 92, 95, 96), transfer stress (97), high fat diet, and fat (16, 38, 66, 95)], that clearly reflect the involvement of *avUCP* in fatty acid β-oxidation and cell metabolism. Moreover, one study reported that a high concentration of chemical stimulus like ammonia can be effective in the expression of 12 energy metabolism-related genes (*avUCP, HK1, HK2, PK, PFK, PDHX, CS, LDHA, LDHB, SDHA, SDHB*, and *AMPK*), in chicken liver. Otherwise, it was reported that ammonia gas resulted in mitochondrial damage, ATPase reduction, and ultimately reduction of energy release in the chicken liver (98).

### 4.6. Pathway crosstalk

Finally, among 11 enriched pathways, interaction of five signaling pathways including MAPK, adipocytokine, mTOR, insulin, ErbB, and GnRH was predicted, indicating a possible combination of positive and negative feedback among pathways to regulate *avUCP* functions. In general, biological pathway crosstalk refers to the different feedback in seemingly distinct pathways (99). Consequently, it seems that maintaining a delicate balance of *avUCP* functions such as lipid metabolism, thermogenesis, response to cold, and response to ROS can occur by crosstalk between involved pathways. Moreover, when a single gene is considered in depth, within a network of genes, all potential regulatory interferences will emerge. Additionally, it is also known that the interaction between pathways can regulate specific gene expression (100, 101).

A panoply of changes in the primary sequence of *avUCPs* can potentially be involved in changes to protein function and expression through alteration of the final structure of the *avUCP* molecule. Accumulation of findings represents *avUCP* as an essential gene for whole-body energy balance, adaptive thermogenesis, and antioxidant defense in birds. This study contributes to a better understanding of *avUCP* characterization, function, and critical signaling pathways for evaluating how it is regulated in avian species exposed to different conditions. Additionally, the present study provides putative functions for *avUCP*s, and indicates some of the genes, pathways, and mechanisms that are involved in fine-tuning mitochondrial oxidative phosphorylation.

In conclusion, we have compared the sequence, structure and physio-chemical properties of *avUCP* in 8 bird species and determine the functional pathways and networks in which *avUCP* is involved. Oxidative stress in birds is known as one of the most energy-demanding events influencing energy expenditure, the balance of detoxification of free radicals, and oxidative phosphorylation, so *avUCP* could be viewed as a significant marker for developing heat-stress-resistant breeds in future genomic selection programmes.

## Data availability statement

The data presented in the study (nucleotide coding sequences and amino acid sequences of avian uncoupling proteins in FASTA format) are deposited at https://figshare.com/ with the accession number https://figshare.com/search?q=10.6084%2Fm9.figshare.20518086 and are also presented in Table 1.

## Author contributions

PD and MG-Z conceived the overall design, undertook the project management, contributed to the interpretation of results, and critically revised the manuscript. PD carried out the analyses and drafted the manuscript. MD, MR, SK, and EE performed the review part of this research, collecting a set of genes used for gene network analysis and provided support in the analyses. JS revised the entire manuscript, contributed to the interpretation of results, and critically reviewed the manuscript. All authors read and approved the final manuscript.
